# Cardiac Positron Emission Tomography Myocardial Perfusion Imaging: Seeing Beyond Perfusion

**DOI:** 10.1055/s-0046-1823006

**Published:** 2026-05-16

**Authors:** Maseeh uz Zaman, Nosheen Fatima, Sidra Zaman, Rabiya Fatima Hashmi

**Affiliations:** 1 Section of NM and PET/CT, Department of Radiology, Aga Khan University Hospital, Karachi, Pakistan; 2 Department of Nuclear Medicine and Theranostic, Dow University of Health Sciences, Karachi, Pakistan; 3 Department of Internal Medicine, MedStar Health, Baltimore, Maryland, United States; 4 Department of Medicine, Liaqat University of Medical and Health Sciences, Jamshoro, Pakistan

**Keywords:** coronary flow reserve, limitations, MPI, myocardial blood flow, PET, SPECT, strengths

## Abstract

Positron emission tomography (PET) myocardial perfusion imaging (MPI) is a highly accurate noninvasive modality for evaluating coronary artery disease due to its excellent sensitivity and specificity, excellent image quality, efficient imaging protocol, and significantly lower radiation exposure. Its ability to absolutely quantify myocardial blood flow (MBF), MBF-R, and left ventricular ejection fraction-reserve also strengthens its diagnostic and prognostic properties and guides physicians about the justification for coronary revascularization. Presence of coronary artery calcification on the CT component indicates atherosclerosis, and its presence with normal PET MPI indicates subclinical nonobstructive coronaries and warrants risk factor medication to avoid future major adverse cardiac events. Metabolic imaging with FDG PET is considered the gold standard for diagnosing hibernating myocardium and helps in the management of sarcoidosis and infection of the cardiovascular system and implanted devices. However, the huge upfront and operational costs are the primary factors for the limited availability of PET MPI.

Myocardial perfusion imaging (MPI) has long been a cornerstone in the evaluation of coronary artery disease (CAD). For decades, single-photon emission computed tomography (SPECT) MPI has been the most widely used modality. While SPECT MPI has proven clinical value, it is inherently limited by its relative perfusion assessment, lower spatial resolution, and susceptibility to attenuation artifacts. 1 Furthermore, balanced ischemia, diffuse CAD, and coronary microvascular dysfunction may go undetected with SPECT MPI. Additionally, longer acquisition times and higher radiation exposure can impact patient comfort and safety (especially in younger populations). 1

However, advancements in imaging technology and a growing emphasis on precision medicine have led to a paradigm shift toward positron emission tomography (PET) MPI. In fact, PET MPI has evolved from a modality focused solely on detecting regional myocardial perfusion abnormalities to a comprehensive tool that provides deep insights into coronary physiology, myocardial viability, and patient prognosis. By going beyond visual perfusion assessment, PET MPI offers quantitative and functional information that provides excellent diagnostic accuracy and clinical decision-making. 2 In this review, we will look into the strengths of PET MPI and factors contributing to making it a highly advanced, multidimensional, and multiparametric imaging modality.

PET MPI demonstrates *excellent diagnostic accuracy* for the evaluation of CAD and is widely regarded as superior to both SPECT and coronary computed tomography angiography (CTA). In the landmark PACIFIC trial (Perfusion Imaging and CT Coronary Angiography with Invasive Coronary Angiography), published in 2017, a head-to-head comparison of PET MPI with SPECT and CTA was performed. 3 A fractional flow reserve <0.80 measured on an invasive coronary angiogram was considered as a cut-off for hemodynamically significant coronary stenosis. The PACIFIC trial found a superior diagnostic accuracy of PET MPI (85%; 95% confidence interval [CI]: 80–90%) than SPECT MPI (77%; 95% CI: 71–83%; *p* = 0.02) and CTA (74%; 95% CI: 70–82%; *p* = 0.75). 3

PET MPI is recognized for its *excellent image quality* , which significantly augments diagnostic confidence in the evaluation of CAD. Indeed, the excellent image quality of PET MPI makes it superior to other noninvasive cardiac imaging modalities. Routine and robust scatter correction and attenuation correction (using CT) contribute to enhancing image quality. 4 Similarly, the spatial resolution of PET tracers is also one of the major contributors [comparatively lower for Rubidium-82 ( ^82^ Rb) due to longer positron range in soft tissue; higher for Ammonia-13 ( ^13^ NH _3)_ and ^18^ F-Flurpiridaz ( ^18^ Flurpiridaz) due to shorter positron range; very high for ^15^ O-Water ( ^15^ O-H _2_ O) as it diffuses freely through the cell membrane]. 4 Furthermore, better myocardial tracer uptake of PET tracers than SPECT agents (adequate for ^82^ Rb; higher for ^13^ NH _3_ and ^18^ Flurpiridaz; significantly higher for ^15^ O-H _2_ O) also contributes toward excellent image quality. These factors are responsible for the high specificity of 89% of PET MPI. 5

PET cardiac tracers have *better pharmacokinetics and a lesser roll-off effect* at high coronary blood flow (except ^82^ Rb—an analogue of potassium like thallium-201, ^201^ Tl). In an ideal scenario, tracer uptake by myocytes would be linear and directly proportional to myocardial blood flow (MBF) across all rates. However, with many currently used perfusion tracers, as blood flow increases significantly during pharmacological stress, the heart muscle's ability to extract the tracer from the blood reaches a saturation point, or “rolls off,” resulting in a nonlinear relationship. 6 This nonlinearity is a major source of error in quantitative MBF analysis, particularly for detecting less severe CAD or microvascular dysfunction, as the actual high flow is not accurately represented by the image signal. The roll-off effect is most pronounced with commonly used SPECT tracers like thalium-201, tetrofosmin, and methoxyisobutyl isonitrile (MIBI), but less pronounced with most PET tracers. ^82^ Rb has the most significant roll-off among the commonly used clinical PET agents, as it is a potassium analog like ^201^ Tl. Its extraction fraction is lower (around 65% at baseline) and drops considerably as blood flow increases and requires scatter correction, which also amplifies noise (moderate flow quantification). 4 In contrast, the extraction fraction is higher for ^13^ NH _3_ (80%) and ^18^ Flurpiridaz (90%), and excellent for ^15^ O-Water (100%), resulting in good, very good, and excellent flow quantification, respectively. 6 The higher extraction fraction and absolute MBF quantification during vasodilator stress ensure a sensitivity of around 90% of PET MPI. 5

The rest-stress PET MPI imaging protocol is *time-efficient* due to the short half-life of PET tracers. A rest and pharmacological stress PET MPI using ^82^ Rb and ^15^ O-water is usually completed within 30 minutes due to the short half-life ( ^82^ Rb: 76 seconds ^, 15^ O-H _2_ O: 2 minutes). However, the rest-stress protocol using ^13^ N-ammonia (half-life 10 minutes) and ^18^ Flurpiridaz (half-life 110 minutes) is completed within 1 and 2 hours, respectively. 4 In contrast, a stress-rest SPECT MPI study using Technetium-99m (half-life 6 hours) labeled Tetrofosmin or MIBI needs approximately 3 to 5 hours to be completed (1- or 2-day protocols). 7

Another strength of PET MPI is the significantly *lower radiation exposure* to patients, health care staff, and family members due to the short half-lives of radiotracers. In 2009, the National Council on Radiation Protection and Measurements revealed that in the United States, the per capita radiation dose from medical procedures had increased nearly sixfold from the early 1980s to 2006. 8 To address this issue, the American Society of Nuclear Cardiology in 2010 released an advisory for nuclear cardiology laboratories to adopt MPI protocols to keep radiation exposure less than 9 mSv (milli-Sievert). 9 Various protocols have been devised to achieve this target, like stress-only imaging, stress-first protocol, avoiding ^201^ Tl, weight-based dosing of SPECT tracer, and use of modern cameras like cadmium–zinc–telluride cameras. However, a study published in 2018 revealed that U.S. laboratories applying for Intersocietal Accreditation Commission accreditation were found to have radiation exposure of 12.8 mSv (665 laboratories) for SPECT MPI, but 3.7 mSv (111 laboratories) for PET MPI. 10 A lower radiation exposure from PET MPI is very important for the younger population undergoing these diagnostic procedures.

Using a vasodilator as stress (adenosine or regadenoson) in PET MPI allows to precisely measure the *left ventricular ejection fraction (LVEF) at peak stress.* The ratio of LVEF at peak stress to rest is called LVEF Reserve (LVEF-R), which has significant diagnostic and prognostic value in CAD. Its diagnostic strength was expressed in a study published in 2007 that a normal LVEF-R (≥5%) has a 97% negative predictive value (NPV) to exclude left main or three-vessel disease (sensitivity 94%; specificity 55%). 11 Its prognostic strength is shown in another study revealing that LVEF-R of 7.2% was found in patients having a low likelihood for CAD, but LVEF-R of 5.2%, or 3.9%, or decline (−2.8%) was found to have an inverse correlation with the Duke Angiographic Jeopardy Score. 12

MBF depends not only on epicardial vessels (microcirculation) but also on small vessels (microcirculation). PET MPI is the gold standard technique for measuring MBF in mL/min/g. The ratio of MBF at peak stress and rest is called the coronary flow reserve (CFR), which has proven its diagnostic and prognostic strength and guides the cardiologist in managing CAD. A study has shown that a normal CFR (>1.93) was found to have 97% NPV for excluding high-risk CAD on angiography. 13 Another study that included more than 12,000 patients revealed that patients with CFR >1.8 had significantly better survival benefits than those with CFR ≤1.8. 14 The same study also revealed that patients with CFR ≤1.8 who had early revascularization (≤90 days) had better survival benefits compared with those who were managed medically. 14 A normal CFR on PET MPI is considered to rule out obstructive CAD and microvascular disease with a high degree of confidence; it also guides cardiologists to avoid revascularization and ensures the adequacy of vasodilator stress.

Attenuation correction of PET images is mandatory, and *low-dose CT images* are used for this purpose. In addition, CT also provides anatomical mapping for functional abnormalities seen on attenuation-corrected PET images. Coronary artery calcification (CAC) seen on CT indicates plaque and coronary artery atherosclerosis. 15 Absence of CAC in a normal PET MPI implies an excellent long-term prognosis. Conversely, the presence of CAC in a patient with an otherwise normal PET MPI result indicates the presence of subclinical nonobstructive coronary atherosclerosis, which is associated with a higher rate of major adverse cardiac events (MACE) depending upon the burden of CAC. 16 These patients will benefit from more aggressive risk factor modification and closer clinical follow-up.

PET MPI (or SPECT MPI) with *myocardial metabolic PET imaging using 18F-fluorodeoxyglucose ( ^18^ FDG)* remains the gold standard for clinical assessment of myocardial viability and is the only imaging modality capable of directly identifying the presence and extent of hibernating myocardium. A perfusion defect seen on PET or SPECT MPI with preserved glucose ( ^18^ FDG) uptake, *perfusion–metabolism mismatch* , is considered the hallmark for hibernating myocardium. 17 ^18^ FDG is also clinically useful for the assessment of cardiac sarcoidosis and is superior for detecting active inflammation, and is the preferred method for monitoring a patient's response to immunosuppressive therapy. With cardiac MRI, it has a complementary role, which is excellent for identifying fibrosis (scarring), and helps in predicting the prognosis. 18 ^18^ FGD PET/CT is also a powerful imaging tool for diagnosing tricky cardiac infections, especially prosthetic valve endocarditis, cardiac device infections (pacemakers, left ventricular assist devices), and culture-negative endocarditis, offering high accuracy where echocardiography fails. 19 ^18^ FDG also plays an important role in the evaluation of vasculitis and vascular graft infections. 19

Despite the above-mentioned strengths of PET MPI, it has several *limitations* related to logistics, tracer physics, and clinical application. The upfront cost for PET/CT scanners and the necessary infrastructure is significantly higher than that for SPECT equipment. Due to the short half-lives of PET tracers, the operational cost for a sustainable supply of PET tracers is humongous, too. The huge upfront and operational costs are the primary factors for the limited availability of PET MPI. 6 ^82^ Rb generators require replacement every 4 to 6 weeks at high costs, while ^13^ N-ammonia and ^15^ O-water typically require an on-site or nearby cyclotron. These are the fundamental factors making it less accessible in rural or underserved areas compared with SPECT. Due to the short half-lives of PET tracers ( ^82^ Rb: 76 seconds; ^13^ N-ammonia: 10 minutes; ^15^ O-water: 2 minutes), they must be injected while the patient is on the scanner bed. This makes physical/exercise stress testing (on a treadmill) nearly impossible, forcing a reliance on pharmacologic stress agents. However, the recently introduced tracer ^18^ Flurpiridaz (half-life: 110 minutes) allows dynamic exercise using a treadmill. The longer positron range of ^82^ Rb can lead to lower spatial resolution and blurry images compared with other PET tracers like ^18^ F. 4 ^82^ Rb, being a potassium analogue, has a lower extraction fraction at high flow rates, which can potentially lead to an underestimation of MBF flow at peak stress (roll-off phenomenon). 4 In PET metabolic imaging using ^18^ FDG (used for viability, infection, or sarcoidosis), high blood sugar levels can interfere with tracer uptake, leading to inaccurate results. 20 Patient movement, including respiratory motion, can cause blurring or displacement of lesions on the scan, leading to potential misinterpretation.

PET MPI is a highly accurate noninvasive modality for evaluating CAD due to its excellent sensitivity and specificity, excellent image quality, efficient imaging protocol, and significantly lower radiation exposure. Its ability to absolutely quantify MBF, CFR, and LVEF-R also strengthens its diagnostic and prognostic properties and guides physicians about the justification for coronary revascularization. The presence of CAC on the CT component indicates atherosclerosis, and its presence with normal PET MPI indicates subclinical nonobstructive coronaries and warrants risk factor medication to avoid future MACE. Metabolic imaging with FDG PET is considered the gold standard for diagnosing hibernating myocardium and helps in the management of sarcoidosis and infection of the cardiovascular system and implanted devices. However, the huge upfront and operational costs are the primary factors for the limited availability of PET MPI.
